# A Life Worth Sustaining? Bestowed Worth and Pediatric Care

**DOI:** 10.1002/hast.70015

**Published:** 2026-03-16

**Authors:** Daniel T. Kim, Xiang Yu

**Keywords:** best interest standard, relational potential standard, bestowed worth, meaningfulness, pediatric decision‐making, medical ethics, parent‐child relationship

## Abstract

*When parents request life‐sustaining treatments for children who suffer from profound neurocognitive disabilities or are at the end of life, the typical ethics advice for clinicians is to accommodate the request. It can be unclear what interests such children have, and being unable to assess those interests, a clinician will tend to honor parents’ requests to continue treatments if the associated pain can be palliated. But how is the clinician not participating in using a profoundly vulnerable child merely to satisfy parental interests? In what sense can their actions be experienced as worthwhile? These circumstances can be morally distressing for clinicians, and recent efforts to justify the practice according to a* relational potential standard *seem problematic. We therefore propose an alternative* meaningfulness standard, *which builds on a notion of* bestowed worth *to explain how a clinician's duty to treat in these cases can be meaningful, and why it need not entail using the child as a mere means*.

## Article

Two recent articles in major bioethics journals have revived a 1984 article by John Arras, “Toward an Ethic of Ambiguity,”^1^ to make a case for a *relational potential standard* in assessing the ethics of continuing life‐sustaining treatments for children with profound, irreversible cognitive impairments.^2^ Written by Aaron Wightman et al. and by Jenny Kingsley et al., the two articles make welcome attempts to address an ethical dilemma that arises frequently in intensive care units and that clinical staffs in those units describe as among the most morally distressing.^3^ Must intensive life‐sustaining treatments continue when the expected outcome for the child is death or, at best, say, a reduced lifespan with profound neurocognitive disabilities, no ability ever to communicate or be aware, and complete dependence on others for activities of daily living? How, if at all, can continued treatment be in such a child's best interest?

Arras and those drawing on his work rightly point out the inadequacies of the best interest standard in such cases and the need to define what constitutes a meaningful human life. This standard, they argue, fails to explain why the life of a child in such circumstances should or should not be sustained because such children lack discernible interests. This shortcoming can be a source of intense moral distress for the clinician who must carry out the aggressive life‐sustaining measures. When a child has no prospect of ever achieving any worthwhile human interests, it is of little comfort simply to say, “Because the parent said so.” It can be difficult to understand the obligation to prolong the child's life, and the aggressive efforts can feel like a meaningless, if not wanton, imposition on a profoundly vulnerable human being. How, after all, is the clinician not participating in using this child as a mere means? Indeed, how can the clinician make sense of their participation in such cases as worthwhile?

As an alternative to the best interest standard, Arras proposes a relational potential standard as a threshold of meaningful life. If a child lacks the potential to attain a basic human good of relationality due to an expected lack of capacity for it, then they should be allowed to die regardless of a parent's wishes. Children with severe and irreversible cognitive disabilities due, for example, to anencephaly or other profound neurological conditions would presumably fall below the threshold. Those who must be continuously sedated at the end of life and cannot come off sedation due to their condition would also likely fit the sort of cases at issue. These are children who lack and are expected always to lack distinctively human interests or capacities, including the capacity to relate to others.

However, in our view, this conclusion does not necessarily follow because a person's interests or capacities do not exhaust the sources of a meaningful life—a life worth sustaining. A life with certain interests can surely be meaningful, as can a life with the capacities to realize those interests. We will show, however, that meaning can also be *bestowed*; a life that is bestowed with a particular significance can have a distinctive worth that should be respected. A certain mode of love—what Nicholas Wolterstorff calls “love as attachment”—imparts worth that inheres in the child: the worth is the child's, and the moral duty is to respect the worth of *their* life, as an end and not a means. Building on this insight, we propose a new *meaningfulness standard* by which clinicians might make sense of their obligations to prolong the life of children who have profound neurocognitive disabilities or are at the end of life.

## In Search of a Threshold of Meaningful Life

Determining a threshold of meaningful life is fraught with moral risk, as it requires quality‐of‐life judgments about what kind of life is worth living. Such judgments can be prone to bias and stigma, and the stakes could not be higher, given that the purpose of the threshold is to help determine which vulnerable child should live or die. A common tendency is to get around the question by focusing on *who* should decide. But, as Arras points out, no matter who is authorized to decide—parent, physician, or ethics committee—they will need to explain the basis for their decision. At the very least, their authority to decide should be constrained by certain ethical limits,^4^ such as not deciding solely to benefit their own interests. The same biases and stigmas that make judgments about a life worth sustaining difficult thus underscore the need for a threshold to guide principled decision‐making.

When Arras sought a threshold in his 1984 article, the Reagan administration had pursued aggressive regulations in the wake of public debates around the case of Baby Doe, who had been born with trisomy 21 (Down syndrome) and trachea‐esophageal atresia. The obstetrician had testified that “the possibility of a minimally adequate quality of life was non‐existent [due to] the child's severe and irreversible mental retardation,” and while other doctors disagreed with this assessment, the parents ultimately exercised their right to refuse surgery for their child—a right that was supported by the courts.^5^ However, the Reagan administration deemed the decision to be discriminatory against those with disabilities, and a month after Baby Doe's death, it enacted federal rules to prohibit future outcomes of this sort.^6^ It rejected “any and all quality‐of‐life judgments—sanctioning nontreatment only for children on the brink of inevitable death.”^7^ Posters were put up in neonatal intensive care units (NICUs) to warn against denying food or other treatments “discriminatorily,” and a toll‐free number and so‐called Baby Doe squads were set up to ensure that perceived violations could be reported and investigated.^8^

The Reagan administration's reliance on the principle of nondiscrimination, however, was not without problems. The principle requires that a child not be denied benefits based on morally irrelevant reasons. So, when deciding whether a child's life should be sustained, factors such as their sex, race, or parent's poverty rightly should not be considered relevant. But, as Arras notes, a child's severe impairment can be morally relevant if, for instance, the child's condition means that continuing treatments would be extraordinarily burdensome for them or cause suffering. The principle thus raises the question, at what threshold is an impairment morally relevant to the decision? Insisting on making no quality‐of‐life judgments does not negate the question. Rather, it effectively presumes that the only relevant threshold is “the brink of inevitable death” and precludes relying on quality‐of‐life judgments that otherwise seem highly relevant.^9^

In 1983, the President's Commission for the Study of Ethical Problems in Medicine and Biomedical and Behavioral Research intervened with the report *Deciding to Forego Life‐Sustaining Treatment*. The report proposed the “best interests of the infant” as the appropriate threshold, as evaluated from “the infant's own perspective”—that is, in terms of what the infant would find beneficial or burdensome, as opposed to what would be convenient to the parents or clinicians.^10^ This was intended to guard against imposing the sensibilities and wishes of “normal adults,” which are frequently prejudicial against the experiences of those with disabilities. The Commission thus concluded that withholding treatments that do not benefit the child's interests is justifiable. The American Academy of Pediatrics has since also elaborated that “when death is very likely and survival would be accompanied by high risk of unacceptably severe morbidity, intensive care is not indicated.”^11^ However, “unacceptably severe” is an evaluative judgment in which parents should be actively involved. The Commission allowed that when parents wish to continue treatments to delay death, the treatments should continue “as long as this choice does not cause substantial suffering for the child.”^12^

Being required to accommodate parental wishes for treatments that do not benefit the child is a paradigmatic source of moral distress for clinicians in intensive care units.^13^ According to Arras, the requirement follows from the application of the best interest standard. This standard cannot constrain parental wishes in such cases because only so much can be known about what a child's interests are. Suppose that “the child suffers no pain. He lies there in his crib, blind, deaf, and uncomprehending. Never having experienced the satisfaction of normalcy, the infant is neither horrified by his plight, nor depressed by … the thought of his impending doom. He just lies there.”^14^ Is it in this child's best interest to die or to live? If this child were suffering due to pain that only death could relieve, then he may have an interest in being pain free and being allowed to die. But pain can often be palliated very effectively, and in the absence of pain, it can be difficult to understand what other interests this child could possibly have, including any interest in dying. Per Arras, the best interest standard thus lacks any basis on which to operate, and the predicament is especially clear when the child is not expected ever to develop distinctive human interests or capacities. As a result, when a parent insists on continuing life‐sustaining treatments, the default decision tends to be “an indiscriminate mandate to treat, to keep alive, that flies in the face of common sense”^15^ and risks treating the child primarily to benefit others’ interests.

Reacting to this circumstance, Arras proposes changing the question. The question in such cases is not simply whether continued life‐sustaining treatments will further the child's best interests, as that cannot be answered. Rather, the inescapable question for whoever must decide is whether—indeed, why—this child's existence is worth prolonging.

## Relational Potential as a Threshold?

Arras argues that we should give up on the best interest standard and adopt a relational potential standard when deciding whether to prolong the life of an infant with profound neurocognitive disabilities or who is at the end of life. He proposes that if a child lacks the ability to be aware of themselves or to relate to others, then withdrawing life‐sustaining treatment from them would be warranted because such a life is no longer worth living.^16^

Wightman et al., who draw on Arras's work, agree on the moral significance of relationships, but they argue that Arras's formulation of the relational potential standard needs to be revised because how society views disability has changed since the time Arras wrote his article. While the push for continuing life‐sustaining treatments for children with profound cognitive impairments used to come from the federal government, often with public support, it now comes more often from the parents. Their relationship with the child is more intimate than that of the state's, which makes a moral difference. Asking if a child can ever participate in a relationship misses the point because the child is seemingly already in one.

Wightman et al. argue that “the caring relationship between parent and child remains morally meaningful even if the child has limited or no observable ability to reciprocate and the relationship may appear one‐directional to an outsider.”^17^ They appeal to care ethics to locate the source of meaning of the child's life in the parent‐child relationship, which is characterized by the norms of emotionally engaged interdependence rather than a rational calculus of each party's competing interests or rights. To be sure, the relationship between a parent and an infant with profound impairments can often look one‐sided, but Wightman et al. insist that this appearance does not mean that the infant is not participating in the relationship. There are other ways in which the child may participate, from cooing and grabbing to touching and being hugged. Or, as Eva Feder Kittay points out, we humans are all dependent to varying degrees, and all our relationships are in fact fundamentally of interdependence.^18^ Wightman et al. conclude that seemingly one‐sided relationships can still be a reason to continue life‐sustaining intervention, “so long as the associated harms of the treatment (primarily pain) to the child are limited.”^19^

Kingsley et al. extend Wightman et al.'s formulation of the relational potential standard to a case involving a critically ill toddler who is at the end of her life. The parents request that the care team not withhold life‐sustaining treatments such as cardiopulmonary resuscitation or intubation, even though the care team thinks that would be contrary to the patient's best interest. Kingsley and colleagues recommend honoring such parental requests “when the child is terminally ill and there is clinical agreement that they are imminently dying,” arguing that, in such situations, “it is ethically permissible to shift from a narrow consequentialist focus (the impact on the child) to a broader one (the impact on the family).”^20^ Defining the best interest of a dying child can be challenging given that the child has a very short future and that there is no agreement on what a “good death” looks like.^21^ In contrast, the death of a child will have lasting impacts on the family, and the family's interests are not easily separated from the child's. Hence, as long as the child's pain and suffering are minimized, Kingsley et al. argue, the interests of the family can be respected.

In another article in this issue of the *Hastings Center Report*, Pierce Randall questions the necessity of the relational potential standard, observing that it seems to collapse into the best interest standard because respecting caring relationships can be understood as contributing to the interests of the child.^22^ In other words, a child's being in a familial relationship has instrumental value because the relationship benefits the child who has it. This is more clearly the case with Arras's formulation of the relational potential standard, which presumes that a capacity for relationships is what makes life valuable, such that the patient's best interest consists in having their potential for a relationship preserved. Wightman et al. and Kingsley et al. are possibly ambiguous on this point, but these scholars seem to regard the relationship as an interest of the child that can be weighed in decision‐making with other interests, such as avoiding pain or restoring bodily functions.^23^ These scholars permit, for example, actions to promote the parent‐child relationship if any discomfort to the child is transient and manageable.^24^ However, if the value of the relationship or of being part of one is derived from the interests that it serves, then the relational potential standard may lose much of its appeal relative to the best interest standard.

Wightman et al. and Kingsley et al. might respond to this objection by arguing that the value of caring relationships cannot be reduced to the interests of each party in the relationship. They suggest that the relationship itself, as a whole, is a good that clinicians have an obligation to preserve. Kingsley and colleagues, for instance, write that “parental requests for invasive or potentially inappropriate therapies at EOL [end of life] do not necessarily reflect an assessment that such treatment will benefit the individual parent or child, but instead that there is inherent value in a nurturing, independent parent‐child relationship.”^25^ The same point is made by Wightman and colleagues, who affirm that the good of a caring relationship “provides strong reason for clinicians to support a parent who chooses to preserve and cultivate such a relationship.”^26^ If the relationship has an intrinsic value that is irreducible to the child's interests, then the relational potential standard would be distinct from the best interest standard.

However, as Randall observes, if the value of caring relationships is independent of the child's interest, then Wightman et al.'s revised formulation of the relational potential standard may run into a second problem: it implies that promoting a familial relationship can be justified even if it does not benefit the child. This approach risks violating what Randall calls an “exclusionary criterion” that enjoys a deep moral appeal in today's clinical practices: namely, medical decisions for patients whose wishes are unknown should be made solely on the basis of the interests of the patient, not on the impersonal value of a familial relationship or the interests of any parties other than the patient, such as the patient's family, the well‐being of the medical team, or overall social utility.^27^ By extending Wightman et al.'s formulation, Kingsley et al.'s work is, we believe, susceptible to the same critique.

In our view, Randall illustrates an important ambiguity within the reformulated relational potential standard. To differentiate their approach from the best interest standard, Wightman et al. and Kingsley et al. at times overstate the independent good of the parent‐child relationship, as though it were self‐justifying. But in fact, they could counter Randall's second critique by insisting that the relationship is not self‐justifying because its good consists in being caring, and an ethics of care can affirm the exclusionary criterion.^28^ A relational potential standard grounded in care ethics supports the need to treat the child as an end insofar as the good of the relationship is understood to be “inclusive of the well‐being of the individuals who are in that relationship.”^29^ However, the problem is that, by avoiding the second critique in this way, it becomes susceptible again to the first critique—namely, the good of the relationship effectively depends on the well‐being interests of the parties involved. In other words, the reformulated relational potential standard tries to have its cake and eat it too.

## Back to the Best Interest Standard?

For his part, Randall defends the best interest standard, arguing that parents should be trusted to determine what is in their child's best interest because they typically care for their child and want to do what is best for them. Parents may not be experts at knowing what is best for their child or what kind of life is worth living, but neither is anyone else. It is unclear that judges, administrators, or doctors would be any better placed to make such decisions. Unless the parent asks the clinician to keep the child alive regardless of how much pain their child might suffer, or something equally unreasonable, the clinician should defer to the parent's judgment about their child's best interest.^30^

However, there are multiple problems with defaulting back to a reliance on the best interest standard. This approach tends to assume that parents are the best placed to evaluate their child's interests, perhaps due to having some privileged access to knowledge about their child's interest that others do not. However, it is unclear whether this is the case, and it frequently is not, especially with infants who are imperiled and have not had a long history with their parents. The care team may often know more than the parents about how a child in the NICU is doing, given the amount of time they spend caring for the child and their expertise in caring for children with similar conditions. Some parents are rarely even seen at bedside because of their other obligations or because they find it emotionally difficult to visit their child.

Reverting to the best interest standard leaves the discussion where it started. Proponents of the relational potential standard are motivated by the concern that it is difficult to know what a child's interests are, if there are any, when they suffer from a profound neurocognitive disability with a grave prognosis or are on life support at the end of life. Defaulting to the best interest standard does not help clinicians or anyone else to make better sense of what the child would find to be in their best interest or offer any basis on which to constrain or support parental preferences. Lacking discernible interests, or a voice, the child effectively risks being treated solely for the sake of others’ interests, in which case the standard violates its own exclusionary criterion.

Specifically, with respect to the parent‐child relationship, relying on the best interest standard fails to recognize that a child whose neurocognitive disability deprives them of any potential for having the capacity to participate in a caring relationship cannot have an interest in it. The standard lacks a clear basis for affirming that the relationship is in the child's best interest and should be sustained or given weight. A capacity for relationship would involve being able to be aware of oneself and others, to take pleasure in another person's company, and to show affection toward others. How can a child who never had or will be able to have these experiences have an interest in them?

To be sure, a proponent of the best interest standard might argue that there are some welfare goods, such as knowledge, achievement, and loving relationships, in which all human beings have an interest regardless of their actual desire or capacity for them. According to objective list theories of well‐being, for example, some things are said to be intrinsically good for a person, in the welfare sense, regardless of whether the person wants or values them.^31^ A person who does not want to be part of a loving relationship or may even be averse to it can, on this view, still benefit from it. It might be argued that if a person can benefit from a relationship without having any pro‐attitudes toward it, then they can also benefit from it even if they do not have the capacity to participate in it. If this is right, then objective list theories of well‐being may be able to explain why a child whose neurocognitive disability deprives them of any potential for relational capacity can still have an interest in it.

However, while objective list theorists think that a person does not need to have pro‐attitudes toward the relationship to benefit from it, they rightly grant that the person must be able to have certain affective, conative, and cognitive abilities to benefit from it. For example, Christopher Rice, a welfare objectivist, argues that loving relationships benefit a person because love is reciprocated in such a relationship. Some intrinsic goods require that a person have certain essential positive attitudes, and in particular, loving relationships “require that a person desire the good of another and take pleasure in that person's company.”^32^ A child whose illnesses deprived them of the ability to have any desires or feel any pleasure could not be considered to be part of a loving relationship, even if they were loved by their parents. Among those who are conscious, Rice thinks that those who are cared for by others or know what's going on with their lives or the world are better off. But again, this benefit precludes a child who is unconscious.

Guy Fletcher similarly argues for an objective list theory that requires a person to be capable of having certain affective, conative, and cognitive abilities to benefit from an objective welfare good.^33^ His view is motivated by Peter Railton's influential claim in the well‐being literature that, for something to be good for a person, it must engage that person in some way.^34^ On Fletcher's account, all intrinsic goods are at least “partly constituted by affective, attitudinal, or volitional states of that person.” For example, for a person to have friendship, they must have “the attitudes of concern and enjoyment that are constitutive of friendship,” though they need not have pro‐attitudes toward friendship itself.^35^ This again means that if a child is incapable of having these states, then they cannot engage with the relationship in a way that can benefit them. Therefore, in our view, appealing to objective theories of well‐being does not entitle proponents of the best interest standard to say that the children in the limit cases that first prompted Arras to propose the relational potential standard can derive interests from being in a loving relationship.

## A Child's Bestowed Worth

Why, then, should clinicians be obliged to prolong the life of a child who lacks any potential for basic human interests or capacities? How would clinicians not be facilitating the use of the child as a mere means? While we agree with our interlocutors’ consideration of the parent‐child relationship, we disagree with their understanding of its ethical significance. The significance of the relationship need not depend on the fact that it is in the child's or parent's interests or that the relationship has an independent worth. Nor does its value turn on a parent's being generally better able to decide what the child's best interests are. Rather, parental love, we contend, can bestow a distinctive worth to a child—which inheres in that child—that clinicians should recognize and respect.

“Bestowed worth,” as Wolterstorff defines it, is an aspectual worth that an entity has by virtue of “standing in some relation to something other than an aspect of themselves.”^36^ Applying this idea to a child, the fact that a child stands in a particular relationship with the parent—say, is loved by the parent—is an aspect of who the child is, and this aspect gives the child a particular worth. In this picture, the child is a good not because she satisfies some desire or need that the parent has or because she participates in a Platonic ideal of a baby or The Good, but simply because she has the property of being loved by her parent.^37^ This fact of being loved is now an aspect of her identity that imparts to her distinctive worth—worth that is not instrumentally reducible to some other good. The fact that she is loved does not make her more useful to the parent or anyone else. Even if it did, it would not detract from the distinctive worth she would still have by virtue of being beloved.

Not any kind of love bestows worth, however. Wolterstorff distinguishes between three types: love as attraction, love as benevolence, and love as attachment.^38^ When one loves in the mode of attraction, one is attracted to a worth that the beloved already has. Far from bestowing worth, the lover expects their own well‐being to be enhanced by engaging with the beloved. One may love the beauty of the aurora borealis in this way, for example, or the bright alertness of a baby's eyes. In contrast, love as benevolence bestows worth, but it does so only by bringing about a positive change in the object that is loved. Benevolently feeding an emaciated pet in need of nutrition enhances their life, but the pet's worth is enhanced not by the feeder's love per se but by the change that their action causes. A stranger could bring about the same change in worth by feeding the pet. Finally, love as attachment is different from either of these modes of love: it bestows worth and does so regardless of any calculus of benefit. One's attachment to the beloved need not be a response to their prior worth. The child may, to the whole world, be ugly and be a physical or emotional burden, but when the parent loves in the mode of attachment, they love regardless of any quality that the child may or may not have or of any satisfaction that the parent may or may not derive from their engagement with her. The child is theirs, and just for that reason, the parent is lovingly attached to her, and this fact imparts to the child a worth that she otherwise lacks.

Does love as attachment really bestow worth? Wolterstorff does not articulate an elaborate philosophical mechanism for how this works but appeals to everyday examples in which we bestow worth in this way and recognize it. For example, people commonly treasure the relics of those they love—deceased parents, grandparents, children, or friends. That ring or this heirloom, picture, or teddy bear may be worn, discolored, broken, or unsellable, but its worth to the owner is still priceless. If asked why the item is priceless, the owner would not appeal to its physical properties, such as its beauty, price, or durability; the owner would simply reason that it belonged to someone they loved. The worth of such a relic is instrumental since it is respected for the sake of the beloved, not necessarily for itself. The relic stands in for the beloved,^39^ and to respect or demean it is to respect or demean the beloved with whom it is associated.

In contrast to relics, a person who is beloved is recognized for who *they* are as a result of their standing in some relationship. To illustrate the idea, Wolterstorff asks us to consider a relationship between a queen and one of her subjects, whom the queen has befriended in the mode of love as attachment. The queen's loving attachment to her friend bestows on the friend a new status, a new worth.^40^ Call the friend Mary. Of course, being the queen's beloved friend gives Mary a status that may be instrumentally beneficial, but the point is that, regardless of those instrumental benefits—even if the queen did not favor her with title or land—Mary would be a friend of the queen. Wolterstorff concedes that how this bestows the friend with a distinctive worth is “mysterious,” but the fact that this is indeed the case can be seen in various indicators. For example, the fact that Mary is beloved by the queen would by itself be sufficient to make her worthy of envy, even without the material or social benefits. Moreover, she would acquire new ways of being wronged. For instance, if someone were to deny that the queen's love for her was in any way noteworthy, they would be demeaning her worth. If someone were dismissive of it, Mary might be angry at being snubbed, and her anger would be understandable. The snub may indirectly demean the queen's worth, but it would also demean Mary, the worth of who *she* is by virtue of her identity as the queen's beloved.^41^

If this example is too monarchical, one might readily imagine, say, a ten‐year‐old boy who is beloved by his parents in the mode of attachment, unconditionally. The fact that he is beloved can be a source of others’ envy. Another child whose parents have died or whose parents don't love her in the same way might say that he is lucky to be so beloved. The boy can also be wronged if his worth as his parents’ beloved child is demeaned. If his classmate were to dismissively mock the importance of his parents’ love for him or to say that he's not in fact beloved, he would be offended. He'd say, “Take that back!”—and not just because his classmate may be insulting his parents. He would be indignant also, or primarily, because he feels directly demeaned. The classmate is demeaning who *he* is—the distinctive identity that he has as a beloved child of his parents.

Along these lines, we argue that the life of a child who is unconscious because they have profound neurocognitive disabilities or are dying but who is beloved in the mode of attachment can have a distinctive worth that clinicians should respect. The obligation is to treat the child in a way that is properly expressive of the child's worth—who the child is—and not for the sake of the parent's interests or the parent‐child relationship. For example, if respecting the worth of the child's life is deemed to require continuing life‐sustaining treatments, then withholding or withdrawing the treatments would not only disrespect the parent who made the judgment but also wrong the child—it would disrespect the child's worth and, in that way, demean her.^42^ Our account of bestowed worth thus offers an important conceptual gain over the relational potential standard: it articulates the significance of the parent's love for the child—its power to bestow worth—without treating the child as a mere means and violating the exclusionary criterion.

To be clear, a child has multiple sources of worth. Parental love is not the only thing that gives a child their worth, nor does it mean that the child who is its object is somehow more valuable overall than other children.^43^ In all fundamental respects, this child shares in the same inviolable dignity that other human beings have.^44^ The basic human worth of children with profound neurocognitive disabilities or who are at the end of life is uncontroversially and widely recognized, and whether a parent loves the child or not has no bearing on her worth qua human being. We take this for granted, and we add only that the parent's love as attachment imparts to the child a *distinctive* worth—within the bounds of her humanity—that would otherwise be absent. Expressions of parental love that violate a child's basic rights would demean the child's dignity as a human being and could not be said to express a love that bestows worth. Put differently, the worth that a parent's love bestows is not mutually exclusive from the worth of a human life that enjoys interests and capacities that are widely recognized as central to it. A child with a capacity to love or reason, or with an interest in being able to exercise those capacities, can indeed have a distinctive worth as a human being.^45^ However, our point is that these do not exhaust the sources of a child's worth. Worth can also be bestowed by being beloved in the mode of attachment. The life of a child who lacks human interests or capacities but is beloved by their parent in the mode of attachment has a distinctive worth that physicians should honor and not demean.

## A Meaningfulness Standard for Clinicians

Can the phenomenon of bestowed worth be operationalized for clinician decision‐making in the pediatric cases at issue? In our view, the worth of a child's life is inseparable from the question of how that life should be treated, that is, respected. With that in mind, we can now propose a meaningfulness standard for clinician decision‐making.^46^

The standard's practical question is this: would prolonging this child's life (a) serve the child's interests or capacities or (b) be properly expressive of the worth of their life? If the answer to this question is yes, then life‐sustaining treatment should be provided, but if the answer is no, then it should not. The clinician's concern in asking this question would be to guard against overtreatment. The question can also be turned into one about *not* prolonging the child's life, in which case, a yes would permit discontinuing treatments, and no would entail seeking to continue it. The clinician's goal in that case would be to guard against undertreatment.

The meaningfulness standard does not do away with the best interest standard but integrates and reframes it. In contrast to the best interest standard or Arras's formulation of the relational potential standard, our proposed standard asks how the distinctive worth of the life of this beloved child should be respected. Basic human interests and capacities can be a part of that evaluation. But a child's distinctive worth as the beloved of a particular parent can be another basis for treatment decisions, especially when the child lacks discernible interests or capacities. This is because questions of worth are not reducible to a calculus based on the best interest standard: a parent's concern to treat their child's life in a way that affirms her unique, immeasurable worth is not exhausted by considerations of what the child might have an interest in.^47^ By the same token, the meaningfulness standard is not restricted to cases of children who lack interests or capacities. While this standard is motivated by the kind of cases that Arras is concerned about—those involving children who lack the ability to be aware of themselves and the ability to relate to others—it has a broader application. It can be used to guide clinicians’ decisions in cases where the child clearly retains interests and capacities.

***Sustaining life as a meaningful act***. That said, the crux of the challenge for the clinician may be how to answer the standard's practical question, and that may be as much an existential concern as it is a puzzle to be solved. Insofar as clinicians care about, or are not indifferent to, the fate of the child, having to continue or withhold life‐sustaining treatments against their judgment about how best to benefit or protect the child can implicate their professional and personal commitments.^48^ Clinicians commonly report such high‐stakes situations as morally distressing,^49^ and repeated distress of this sort has been linked to burnout.^50^ The outcome thus matters to the clinician given their agency as a participant in the situation. From their standpoint, the following may be at stake: can my participation in prolonging the life of this profoundly vulnerable child with a grave prognosis be understood as a meaningful act? The concern behind this question need not be with *meaning* in some cosmic sense—the meaning of life—but may be with what Susan Wolf terms “meaning *in* life.”^51^ The appeal is to the need that we have as human beings to be fulfilled—to know that what we are doing is meaningful.

According to Wolf, for an action or project to be meaningful to one, it must in some way be fulfilling *and* worthwhile. She calls this a “bipartite view” of meaning that conjoins two popular views on the question: “it doesn't matter what you do with your life as long as it is something you love” (the fulfillment view) and “in order to live a truly satisfying life one needs to get involved in something ‘larger than oneself’” (the larger‐than‐oneself view).^52^ Each view captures a valid insight but is incomplete without the other. Meaningful activities are subjectively fulfilling, arising from doing what one loves or cares about. But if the project is objectively pointless—for example, Sisyphus's act of rolling a boulder up a hill only for it to roll back down, and doing that for eternity—then few would call that meaningful. Even if Sisyphus were said to enjoy doing it, most people would be reluctant to say that his feeling of fulfillment alone would then make the task meaningful.^53^ Most would instead say that Sisyphus is deluded. Rather, to be meaningful, the project also needs to be objectively so, involving something that others do or could later recognize and value. Per Wolf, it needs to involve “something *other* than oneself—that is, … something the value of which is independent of and has its source *outside of* oneself.”^54^

For a physician, who swears a public oath on their personal honor that “the health and well‐being of my patient will be my first consideration,”^55^ caring for a vulnerable child is typically both subjectively fulfilling and objectively worthwhile. However, the issue is whether sustaining the life of a child with profound and permanent cognitive impairments is in fact worthwhile—whether this life has a worth beyond the subjective whims of the parent or clinician. By showing that there is such a thing as bestowed worth, we have argued that, depending on the circumstances, the act can have this worth. The love of a parent in the mode of attachment bestows a worth that is of the sort that can be recognized and respected by others. It is uncontroversial to say that this child is a human being and deserves to be treated as any other child would be treated. But in addition, a child's life can be further distinguished in being loved by their particular parent, and respecting its worth may entail different actions depending on what that requires. The distinctive worth of a particular child's life may require sustaining it in a way that respecting another child's life would not.

Our claim is not that the child's life has a value that is radically objective—an in‐and‐of‐itself value that persists independently of a parent's or clinician's valuation of it. This sort of worth, as Wolf points out, is sometimes associated with Plato or more recently with G. E. Moore.^56^ On such a view, one is either necessarily and everywhere right about the worth of the life being sustained or not. However, it is doubtful that the truth of this sort of valuation could ever be determined with certainty. The worth of a child's life is not a brute fact, a thing that exists “out there” to be observed, measured, and tested scientifically. At best, a scientific method can survey some people's evaluations of that worth, but that is not the same as measuring it directly.^57^ The worth of a child's life is in this way inextricable from people's interpretation and valuation of it.

Rather, our claim of objectivity is quite modest: the determination of worth of a child's life is not reliant solely on their parent's subjective whims.^58^ To be sure, what it means to treat a child's life with respect, in accordance with their distinctive worth, may finally depend on their parent's judgment. This would be in keeping with our society's well‐established ethical and legal deference to parental authority^59^ and, in our view, with the role of parental love as attachment in distinguishing the worth of a child's life. However, parents do not decide in a social vacuum, and they rarely confront decisions about life‐sustaining treatment for their child with ready‐made answers.^60^ A parent who initially insisted on continuing such treatments may wake up one day and realize that their desire to sustain their child's life is mistaken and that continuing treatments would not be meaningful. The thought would not necessarily be that the child's life is now worth less but that forgoing the treatments would be a more adequate way to respect that worth. Moreover, such a change in judgment would typically be experienced as a move in the right direction, that is, toward a decision that is less confusing or contradictory and is clearer or more fitting in some way. Indeed, parents rarely experience their change in decision as arbitrary or want to explain it that way.

A change in judgment of this sort would constitute what Charles Taylor calls “moral growth”—a growth toward a more perspicuous understanding of what is otherwise unclear, confusing, or error prone.^61^ This experience of growth is often what can ground confidence in a decision. To be sure, the growth may be false or illusory. But Taylor's point is that the argument over its validity would necessarily be resolved not by appeal to some absolute criterion but by a new and more adequate interpretation of our lives.^62^ In that sense, the growth is necessarily provisional; it relies on the “best account” that can be given of the situation and what matters in it. Taylor calls this the “BA principle”: it assumes that any account we can give of something like the worth of a child's life is valid “unless and until” it can be replaced with “more clairvoyant substitutes.”^63^

In our view, such experiences of growth are indeed common, and the potential for this growth can be why parents should be given the time, space, information, and support needed to reach an end‐of‐life decision for their child. The aim in these decision‐making processes is rarely, if ever, to find out what decision is objectively “correct,” but neither do we expect the decision to be arbitrary. Per Taylor, the aim should be to decide based on the best account that can be given for a situation and what matters in it. To that extent, a subject‐independent truth or reality must be posited or at least not denied. It must be presupposed that the child's life has some subject‐independent worth about which a parent can be mistaken or that they can rightly reevaluate.^64^ One's experiences of moral growth, and indeed, one's expectations for it, would otherwise be incoherent except as an illusion.

In other words, working to sustain a child's life can be meaningful for the clinician insofar as they are not merely acquiescing to the subjective, unaccountable whims of a parent. A clinician can engage parents in discussion, supporting their decision‐making process, helping to clarify their thinking and appreciation of relevant facts and values, and making recommendations. Indeed, moral growth is possible for the clinician as well. A skeptical physician, for example, may see the deep bond that the parent has with the child and, in time, recognize that perhaps the parent is right to value the child's life in that way. In this way, our proposal both grants that the parent's love as attachment gives the child's life a distinctive worth and maintains the possibility of a shared moral growth toward a more perspicuous account of that worth and how it should be respected.

***A practical decisional process***. In the practice of decision‐making, our meaningfulness standard can thus recommend a shared decision‐making model in which “doctors help parents discern their own values and ethical commitments as they face an unanticipated situation and a series of life altering decisions.”^65^ According to John Lantos, the physician's goal amidst disagreement should be “value clarification,” which departs from traditional approaches in which doctors think of themselves as “information providers” who engage primarily to offer the relevant medical facts. Value clarification involves creating a safe space and following four steps: acknowledge emotion (saying, for example, “How are you doing right now?” or, “Tell me how you're feeling.”); elicit hopes, fears, goals, and values (with, for instance, “What are you hoping for?,” “What are your fears?,” “Do you regret anything that's been done?”); reflect back parents’ views (with language like “So, what I'm hearing you say is …”); and move toward a shared decision (with, for example, “We face some decisions here … ”).^66^ The process may then need to circle back to the first step and continue over time, until a shared decision is finally reached.

In this process, the idea of bestowed worth might prompt the following additional questions when eliciting parental hopes, fears, goals, and values: “We are discussing the life of your beloved child. How can we respect who she is?” or “How can we help you honor your child's life?” These questions do not directly address whether the parent loves the child in the mode of attraction, benevolence, or attachment. Most parents in fact love their children, and the point is not necessarily for clinicians to interrogate parents to judge the quality of their love. But neither should clinicians simply defer to the loving parent by asking only, “Should we prolong your child's life?” Shared decision‐making is instead a process. The doctor's role is not simply to give parents medical information and wait for a decision but to help them clarify their values and how those values bear on the decision that must be made for their child.

Such questions as we propose would be a way to prompt parents to think not primarily in terms of the child's interests or capacities but in terms of the child's status as their beloved child and what it would mean for the team to respect who this child is. Some parents may conclude that honoring who their child is requires advocating for her life and continuing life‐sustaining treatments to affirm its worth. Other parents may conclude that making memories with their child to celebrate her brief life before allowing her to die is what it means to honor her worth. Either way, the clinician's goal would be to work with the parents toward a shared understanding of how to recognize and respect the worth of their beloved child, as an end rather than as a means.

***Limits of parental discretion***. Our account of the meaningfulness standard may raise concerns about whether it is too permissive. However, the parental love as attachment on which it relies imposes crucial ethical constraints. The standard entails that parents should act out of that love.

For one, in line with the best interest and relational potential standards, the harm principle is important. But it is important not because the distinctive worth of the child should be weighed against, say, her interest in not being harmed—as though in a calculus based on the best interest standard—but because the pain and suffering of the child would otherwise transform the worth that a loving relationship bestows. Insisting on life‐sustaining interventions that cause pain or suffering that is unrelenting and unresponsive to exhaustive palliative measures cannot be interpreted as loving. Far from imparting distinctive worth, such an action would demean the basic worth that the child has as a human being. As we noted above, the distinctive worth bestowed by parental love does not obviate the child's fundamental human worth, which requires that they not be subjected to cruel, inhuman, or degrading treatment.^67^ Hence, not causing wanton or undue harm is a principle that must continue to be honored on a meaningfulness standard.

Second, a parent's self‐interested bestowal of meaning would not work, as it cannot bestow worth. Whether the self‐interest is rooted in their own guilt, shame, or grief or other thought of psychosocial or material gain, a parent who relates to their child with purely self‐interested motives would demean their worth rather than distinguish it. The parent would be disrespecting the child's human dignity by treating her only as a means to their own ends. To be sure, this constraint on parental discretion is practically challenging for care teams to apply because it can be difficult and dangerous to accuse a parent of self‐interested decision‐making. It is often hard, if not impossible, to know parental motives, and to venture a guess is to risk bias and prejudice, overt or implicit. Nonetheless, the shared decision‐making model can be an important process in which parents reflect on and clarify their motives and whether they are truly honoring their child's life. Moreover, in the worst‐case scenario, abuse or neglect are well‐established ethical and legal constraints on parental prerogatives and are risks about which clinicians should be vigilant.^68^

In addition, parental discretion can be limited by legitimate societal constraints. As argued above, parental discretion over what the distinctive worth of their child's life entails is accountable to a “best account” of that worth. It should be one that others do or could later recognize and value. Debates and disagreements about that account are inevitable, and ideally, they can be resolved over time in processes of shared decision‐making and what Taylor called “moral growth”—at individual, institutional, or societal levels. But if a relevant disagreement cannot be resolved in a timely manner, physicians and other clinicians should not feel compelled to provide inappropriate treatments that violate existing medical or legal standards.

Consider the case of Jahi McMath. Jahi was a thirteen‐year‐old girl who suffered hemorrhagic complications a few days after oropharyngeal surgery and was declared brain dead on December 12, 2013, in Oakland, California. The parents refused to accept that Jahi had died, mistrusting the clinical team and pointing to the occasional movements in her arms, ankles, and hips. The family contended that Jahi did not receive “the treatment she deserved,” potentially because she was Black. Or, as the family's pastor put it, “Is not Jahi worthy of the highest amount of medical treatment?”^69^ The meaningfulness standard validates the importance of Jahi's parents’ concerns, namely, the need to honor the distinctive worth of her life. However, given the brain‐death diagnosis at the time, the ethics committee at the California hospital was not wrong to conclude that continued life‐sustaining treatments for a deceased patient would be medically inappropriate since “no conceivable goal of medicine … can be achieved,”^70^ including that of sustaining life. If Jahi was dead, prolonging treatments needlessly would violate her body and demean, rather than honor, its worth as that of a deceased human being.

That said, a crux of this case is whether Jahi was in fact dead and how death in such cases should be determined. Brain death is a well‐argued and widely accepted criterion for death, but it nonetheless has a controversial social history and utility, and it is not universally accepted as the “best account” of death. Having been denied their request to continue life‐sustaining treatments in California, the parents moved Jahi to a hospital in New Jersey, where families can reject the brain‐death standard on religious grounds. Jahi's parents also questioned whether she was in fact brain dead, and indeed, according to a subsequent detailed medical case report, the brain‐death diagnosis seems to have been a rare case of a false positive.^71^ Be that as it may, five years after her brain‐death diagnosis, Jahi's heart stopped from abdominal complications on June 18, 2018. So, were her treatments in New Jersey up to that time appropriate? A meaningfulness standard, in our view, can affirm the meaningfulness of the New Jersey clinicians’ efforts to continue to treat Jahi. Within the societal bounds of how death should be determined—and setting aside for the sake of argument whether New Jersey is in fact right to allow this discretion—we would accept that those clinicians provided meaningful care that sought to honor who Jahi was, that is, her distinctive worth as a *living* and beloved child of her parents.

Another constraint on parental discretion is that a child's distinctive worth cannot be used to support decisions that violate legitimate societal prohibitions against discriminatory treatment. A parent cannot, for example, appeal to a child's distinctive worth to make claims on scarce medical resources that violate just rules of fairness to other children. Now, one might turn this constraint around to object that the meaningfulness standard itself unduly favors children who, like Jahi, have parents who love them in the mode of attachment, such that love as attachment cannot be a determinate basis for pediatric care. However, unlike race or sex, a child's parents and the particulars of the parents’ values constitute a morally relevant difference. Within the limits of their discretion, parents are allowed to make different value‐based decisions for their children, and the differing actions and outcomes that result from those choices are not necessarily unjust. Again, as argued above, every child is a human being and should be treated in accordance with their worth as such—whether they are a ward of the state or of loving parents. Their human dignity is a baseline equality shared with all. But within the bounds of what that dignity requires, a particular parent's love for their child can impart a distinctive worth on that child's life, and respecting that particular worth may rightly entail a different decision or treatment course.

## The Meaningfulness Difference

The proposed meaningfulness standard does not necessarily lead to different guidance than current recommendations about the care of children with profound neurocognitive disabilities or who are on life support at the end of life. However, the rationale for what must be done is critical to the ethical lives and commitments of clinicians, and this is where the best interest standard and the relational potential standard fall short. The former is silent on the question of whether continuing life‐sustaining treatments is in the interest of these children, while the latter risks either collapsing into the best interest standard or subordinating the child's good to that of a parent‐child relationship.

Our meaningfulness standard—by building on an account of bestowed worth—thus explains why a clinician's obligation to continue life‐sustaining treatments need not entail using the child as a means. It does so, moreover, to enable clinicians to make sense of the worth of the life that they are sustaining and, hence, of the meaningfulness of their obligations.

## Acknowledgments

This article benefited greatly from the feedback of several scholars, in particular, Pierce Randall, Samuel Reis‐Dennis, Wayne Shelton, and anonymous reviewers of this journal. We also thank colleagues at the Journal Club of the Alden March Bioethics Institute and the Well‐Being Working Group organized by Anthony Kelley and colleagues for their invaluable comments and probing questions.
